# Metformin Prevents Cocaine Sensitization: Involvement of Adenosine Monophosphate-Activated Protein Kinase Trafficking between Subcellular Compartments in the Corticostriatal Reward Circuit

**DOI:** 10.3390/ijms242316859

**Published:** 2023-11-28

**Authors:** Rachel Aruldas, Laura Buczek Orenstein, Sade Spencer

**Affiliations:** 1 Department of Pharmacology, University of Minnesota, Minneapolis, MN 55455, USA; aruld004@umn.edu; 2 Graduate Program in Neuroscience, University of Minnesota, Minneapolis, MN 55455, USA; lauraorenstein@gmail.com; 3 Medical Discovery Team on Addiction, University of Minnesota, Minneapolis, MN 55455, USA

**Keywords:** cocaine, sensitization, adenosine monophosphate-activated protein kinase, metformin

## Abstract

Repeated cocaine exposure produces an enhanced locomotor response (sensitization) paralleled by biological adaptations in the brain. Previous studies demonstrated region-specific responsivity of adenosine monophosphate-activated protein kinase (AMPK) to repeated cocaine exposure. AMPK maintains cellular energy homeostasis at the organismal and cellular levels. Here, our objective was to quantify changes in phosphorylated (active) and total AMPK in the cytosol and synaptosome of the medial prefrontal cortex, nucleus accumbens, and dorsal striatum following acute or sensitizing cocaine injections. Brain region and cellular compartment selective changes in AMPK and pAMPK were found with some differences associated with acute withdrawal versus ongoing cocaine treatment. Our additional goal was to determine the behavioral and molecular effects of pretreatment with the indirect AMPK activator metformin. Metformin potentiated the locomotor activating effects of acute cocaine but blocked the development of sensitization. Sex differences largely obscured any protein-level treatment group effects, although pAMPK in the NAc shell cytosol was surprisingly reduced by metformin in rats receiving repeated cocaine. The rationale for these studies was to inform our understanding of AMPK activation dynamics in subcellular compartments and provide additional support for repurposing metformin for treating cocaine use disorder.

## 1. Introduction

Repeated exposure to cocaine and other drugs of abuse enhances behavioral and neurochemical responses upon successive experiences [1]. This process, termed sensitization, is often measured using locomotor activity [2]. A single cocaine injection reliably augments locomotor activity, and multiple cocaine injections progressively facilitate that response, producing an effect that can be quite enduring [3,4]. Sensitization is studied in animal models as a simple phenomenon thought to have predictive relevance for some aspects of addiction, including drug craving and relapse [1]. Such repeated cocaine pre-exposure facilitates the later acquisition of cocaine self-administration, but the development of locomotor sensitization is not necessarily required for the expression of a sensitized response to the rewarding effects of cocaine [5,6]. The reverse is also seen with rats given extended access to cocaine self-administration, demonstrating a dose-dependent increase in sensitivity to the psychomotor activating effects of cocaine compared to their short-access counterparts [7]. Cocaine sensitization can also augment the motivational influence of a reward-predictive cue for a non-drug reward [8]. Importantly, the occurrence of behavioral and physiological sensitization has similarly been reported in humans [2,9]. 

Behavioral sensitization is driven by neural plasticity, measured in multiple nodes of the corticostriatal circuitry, including the medial prefrontal cortex (mPFC), dorsal striatum (dStr), and nucleus accumbens (NAc) [1]. Cocaine sensitization is associated with increased extracellular dopamine release in the NAc upon a subsequent cocaine challenge [10,11]. Cocaine challenge following sensitization likewise increases extracellular glutamate in the dStr and NAc [12,13,14], which includes enhanced glutamatergic input from the PFC [15]. The molecular mechanisms underlying this enhancement of neurotransmission remain incompletely understood, although numerous cellular adaptations contributing to the expression of behavioral sensitization have been identified [16,17]. For example, mammalian/mechanistic target of rapamycin (mTOR) signaling is required for the expression of cocaine-induced locomotor sensitization through a mechanism that involves the trafficking of GluA2 AMPA receptors in the NAc [16]. mTOR is a tyrosine protein kinase that regulates neurodevelopment, synaptic plasticity, and memory [18]. Interestingly, mTOR signaling shows bidirectional regulation with that of another fundamental cellular energy regulator, adenosine monophosphate-activated protein kinase (AMPK) [19,20,21]. AMPK directly and indirectly inhibits mTORC1 activity [19,20], and mTORC1 negatively regulates AMPK in a regulatory feedback loop [21]. AMPK signaling is thus emerging as an important counterbalance to mTOR, worthy of additional study.

AMPK is a heterotrimeric protein consisting of beta (β1 or β2), gamma (γ1, γ2, or γ3), and catalytic alpha (α1 or α2) subunits, the latter of which, when phosphorylated, significantly increases its enzymatic activity [22]. AMPK is critical for maintaining cellular energy homeostasis in the periphery and central nervous system under normal physiological conditions [22,23]. AMPK is also an emergent therapeutic target for numerous chronic diseases, including neurodegeneration and substance use disorders [24,25]. In rats, acute cocaine injections increased phosphorylated AMPK (pAMPK) in the PFC but decreased pAMPK in the dStr [26]. Chronic cocaine injections producing behavioral sensitization amplified this effect [26]. In contrast, pAMPK was reduced in the NAc core following cocaine self-administration and extinction [27] and it was reduced in the NAc shell following abstinence from cocaine self-administration [28]. Moreover, manipulation of AMPK activity in the NAc core or shell reduced cue-induced cocaine seeking and diminished cocaine reinforcement, respectively [27,28]. These data overall demonstrate a role for AMPK in regulating cocaine reward. 

AMPK is indirectly activated by the type II diabetes drug metformin, which promotes phosphorylation of a regulatory site on its alpha catalytic subunit [29,30]. Metformin is a commonly prescribed oral antihyperglycemic drug from the biguanide class [31]. With respect to diabetes, metformin lowers blood glucose and increases insulin sensitivity, although its precise mechanisms of action remain incompletely understood [29,30,32,33]. Recent studies support the idea that metformin may provide a novel therapeutic strategy for the management of neuropsychiatric diseases like substance use disorders [24,34,35]. Introducing metformin to the NAc core decreased cue-induced cocaine seeking in male and female rats following self-administration and extinction [36], in line with the prior research involving direct manipulation of AMPK with viral or other pharmacological tools [27,28]. Moreover, additional preclinical literature indicates that metformin may reduce symptoms of withdrawal from another psychostimulant drug, nicotine [37]. Furthermore, human and animal studies suggest that metformin mitigates some of the cardiovascular and cerebrovascular risks associated with nicotine exposure [38,39]. There is increasing excitement related to repurposing metformin to address cocaine or other substance use disorders, given its classification as both safe and inexpensive [31,35].

This study aimed to determine the impact of cocaine sensitization on total and pAMPK protein levels in synaptosomal and cytosolic protein fractions of the mPFC, dStr, and NAc of rats. We examined AMPK levels in separate functional subcellular domains, given that cocaine sensitization is known to impact the trafficking of other proteins involved in sensitization, including AMPARs [16]; furthermore, AMPK activation can increase AMPAR expression [40]. Most importantly, the kinase activity of AMPK in cortical tissue is enriched in both the nuclear and synaptosomal fractions compared to some kinases that show preferential activity in one or the other domain [41]. Here, we present evidence of specific changes in subcellular AMPK activity in relation to acute and repeated sensitizing cocaine injections. An additional objective was to determine the behavioral and molecular effects of metformin pretreatment on cocaine sensitization. In this regard, we demonstrated the suppressive effect of metformin pretreatment on the acquisition of cocaine locomotor sensitization and related changes in AMPK expression. Overall, these studies add to our knowledge of the molecular mechanisms underlying psychomotor sensitization to cocaine and provide support for metformin as an intervention to suppress this form of neuroplasticity. 

## 2. Results

### 2.1. Cocaine Locomotor Sensitization 

**Figure 1:**
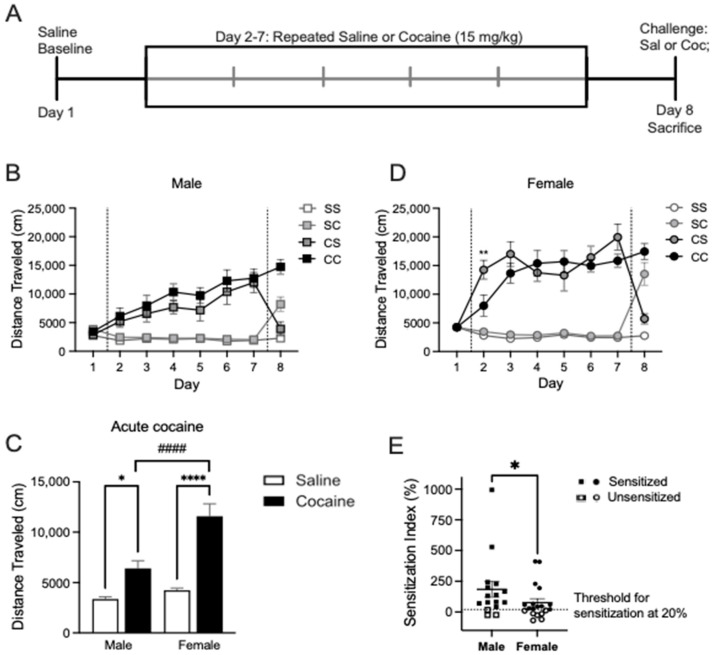
Experiment 1 timeline and behavioral data. (**A**) Timeline of injections followed throughout ex-periment 1. (**B**) Distance traveled (cm) by male rats in the groups saline–saline (SS), saline–cocaine (SC), cocaine–saline (CS), and cocaine–cocaine (CC) on days 1–9. Two-way RM-ANOVA showed a main effect of treatment F_3,20_ = 22.81, *p* < 0.0001, a main effect of day F_3.72,74.49_ = 9.419, *p* < 0.0001, and a treatment x day interaction F_21,140_ = 6.809, *p* < 0.0001. (**C**) Distance traveled (cm) by female rats in groups SS, SC, CS, and CC on days 1–9. Two-way RM-ANOVA showed a main effect of treatment F_3,21_ = 56.70, *p* < 0.0001, a main effect of day F_3.67,77.22_ = 13.35, *p* < 0.0001, and a treatment x day interaction F_21,147_ = 14.56, *p* < 0.0001. ** *p* < 0.01 comparing rats in groups CC and CS on day 2 by Dunnett’s post hoc testing. (**D**) Acute cocaine increased locomotor activity meas-ured as distance traveled by male and female rats. * *p* < 0.05, **** *p* < 0.0001 comparing acute sa-line to acute cocaine. #### *p* < 0.0001 comparing cocaine-treated males to cocaine-treated females. (**E**) Sensitization index of male and female rats exposed to chronic cocaine revealed a significant difference between all male and female rats (* *p* = 0.034).

The rats in experiment one were given injections following the timeline depicted in Figure 1A. To qualify as having demonstrated sensitization to cocaine, there was a requirement for locomotor activity to be increased by ≥20% from the first to the last cocaine injection. The majority of the rats met this criterion, but three males and seven females were excluded from the analysis of protein expression due to a failure to reach this threshold (Figure 1B). Additionally, one male and two female rats in the saline–cocaine (SC) group failed to show increased locomotion above a conservatively set criterion of a ≥30% increase in response to acute cocaine injection and were likewise excluded from the protein analysis. 

When including all subjects, we found a sex difference in the sensitization index (two-tailed Mann–Whitney test performed given a non-Gaussian data distribution, *p* = 0.034; males: 184.7 ± 63.43 and females: 76.07 ± 31.68), but among sensitized rats, this was reduced to a trend level (*p* = 0.068; males: 229.6 ± 72.69 and females: 133.4 ± 41.76) (Figure 1B). The data presented in Figure 1C–E reflect the behavior of only those rats included in the downstream examination of protein level changes; the behaviors and analyses for all subjects (included + excluded) are presented in Supplemental Figure S1, highlighting individual and sex differences in the locomotor responses. In line with previous studies [42], female rats showed higher levels of locomotor activation in response to acute cocaine compared to males (Figure 1C; two-way ANOVA main effect of treatment F_1,34_ = 59.05, *p* < 0.0001; main effect of sex F_1,34_ = 14.46, *p* = 0.0006; sex x treatment interaction F_1,34_ = 10.14, *p* = 0.0031; Tukey’s post-test *p* < 0.0001 male vs. female cocaine). 

No baseline sex differences in locomotor activity were measured with acute saline habituation on day 1 (Figure 1C; Tukey’s post-test *p* = 0.4013 male vs. female saline). Locomotor activity was recorded following each daily injection across all four treatment groups (Figure 1D,E). In males, two-way RM-ANOVA showed a main effect of treatment F_3,20_ = 22.81, *p* < 0.0001, a main effect of day F_3.72,74.49_ = 9.419, *p* < 0.0001, and a treatment x day interaction F_21,140_ = 6.809, *p* < 0.0001 (Figure 1D). Dunnett’s post hoc comparisons illustrated within-subjects effects across time indicative of psychomotor sensitization, with repeated cocaine administration increasing the distance traveled from day 2 to days 4 and 7 in the cocaine–saline group (*p* = 0.014 and *p* = 0.013). Similarly, in the cocaine–cocaine group, locomotion increased from day 2 to days 6, 7, and 8 (Dunnett’s post hoc: *p* = 0.0067, *p* = 0.0281, *p* = 0.0031). In females, two-way RM-ANOVA showed a main effect of treatment F_3,21_ = 56.70, *p* < 0.0001, a main effect of day F_3.67,77.22_ = 13.35, *p* < 0.0001, and a treatment x day interaction F_21,147_ = 14.56, *p* < 0.0001 (Figure 1E). Dunnett’s post hoc comparisons illustrated within-subjects effects across time indicative of psychomotor sensitization with repeated cocaine administration increasing the distance traveled from day 2 to day 7 in the cocaine–saline group (*p* = 0.0163). Similarly, in the cocaine–cocaine group, locomotion increased from day 2 to days 3, 4, 7, and 8 (Dunnett’s post hoc: *p* = 0.0146, *p* = 0.0149, *p* = 0.041, *p* = 0.0013). Overall, we found that the acute locomotor response to cocaine was larger in female rats, but repeated cocaine injections tended to produce greater psychomotor sensitization in male rats.

### 2.2. Effects of Cocaine Sensitization on AMPK Protein Levels

**Figure 2:**
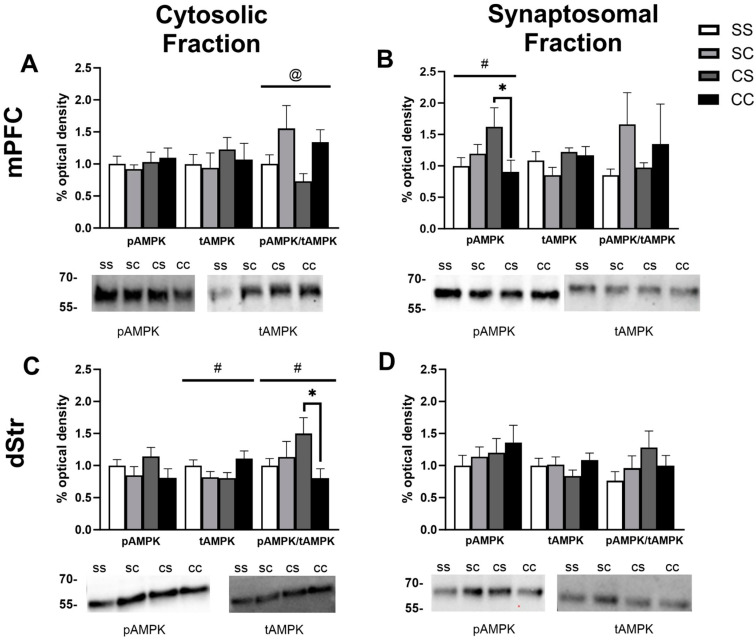
AMPK levels in the mPFC and dStr of rats in experiment 1. Representative bands and optical densities of pAMPK, tAMPK, and pAMPK/tAMPK in the saline–saline (SS), saline–cocaine (SC), cocaine–saline (CS), and cocaine–cocaine (CC) groups. This order is maintained in the graphs and the bands presented in the representative images. Western band cutouts are from 75 kDa–55 kDa to display the pAMPK and tAMPK levels. Protein levels were measured in the cytosolic (left; (**A**,**C**)) and synaptosomal (right; (**B**,**D**)) fractions of the mPFC (top; (**A**,**B**)) and dStr (right; (**B**,**C**)). Two-way ANOVAs were performed to assess the impact of the initial treatment and challenge injections on protein levels. The main effects of challenge injections (@) and the interaction between chronic and challenge injections (#) are denoted in each brain region and subfraction. Sidak’s post hoc tests were performed to identify differences among treatment groups. (**A**) In the cytosol of the mPFC, there was a main effect of the challenge injection on pAMPK/tAMPK (F_1,37_ = 6.580, *p* = 0.0145). (**B**) In the synaptosome of the mPFC, there was a significant interaction between the repeated treatment and the challenge injection when looking at pAMPK (F_1,41_ = 5.07, *p* = 0.0298). * *p* < 0.05 when comparing CS and CC. (**C**) In the cytosol of the dStr, there were significant interactions between the treatment and challenge injections for tAMPK (F_1,40_ = 5.947, *p* = 0.0193) and pAMPK/tAMPK (F_1,40_ = 5.106, *p* = 0.0294). * *p* < 0.05 when comparing pAMPK/tAMPK between CS and CC. (**D**) No main effects were found in the synaptosome of the dStr.

The effects of acute or repeated cocaine treatment on levels of phosphorylated AMPK (pAMPK at Thr-172) or total AMPK (tAMPK) were measured individually in the cytoplasmic (S2) and synaptosomal (P2) subfractions from four brain regions: mPFC, dStr, NAcC, and NAcS. Based on the experimental design, we performed two-way ANOVAs to assess the effects of the initial repeated injections and the final challenge injection, resulting in the four treatment groups. In the cytosolic fraction of the mPFC, we observed a main effect of challenge injection (F_1,37_ = 6.580, *p* = 0.0145) such that rats last receiving a cocaine injection tended to show higher levels of pAMPK/tAMPK independent of whether they experienced prior daily saline or cocaine injections (Sidak’s multiple comparisons: *p* = 0.1594 for SS vs. SC, *p* = 0.1403 for CS vs. CC; no main effect of treatment, *p* = 0.2948) (Figure 2A). No group differences emerged for the individual pAMPK or tAMPK proteins. In the synaptosomal fraction of the mPFC, there was a significant interaction between the repeated treatment and challenge injection (F_1,41_ = 5.07, *p* = 0.0298), but no main effects of either treatment or challenge (*p* = 0.4119 and *p* = 0.2009, respectively) for levels of pAMPK (Figure 2B). We observed a difference between repeated cocaine-treated rats with acute saline versus acute cocaine challenge (CC higher compared to CS, *p* = 0.0298) and a trend toward an overall increase in pAMPK associated with repeated cocaine treatment and short-term withdrawal (SS compared to CS, *p* = 0.0727). Levels of tAMPK and the ratio of pAMPK/tAMPK remained unchanged in the P2 region of mPFC (Figure 2B). In the cytosolic fraction of the dStr, we found significant interactions in our analysis for tAMPK (F_1,40_ = 5.947, *p* = 0.0193) and pAMPK/tAMPK (F_1,40_ = 5.106, *p* = 0.0294) (Figure 2C). Sidak’s post hoc testing showed a trend toward increased levels of tAMPK in CC vs. CS (*p* = 0.0696), while pAMPK/tAMPK was lower in CC vs. CS (*p* = 0.0201). In the synaptosomal fraction of the dStr, treatment had no impact on the levels of pAMPK, tAMPK or their ratio (Figure 2D).

**Figure 3:**
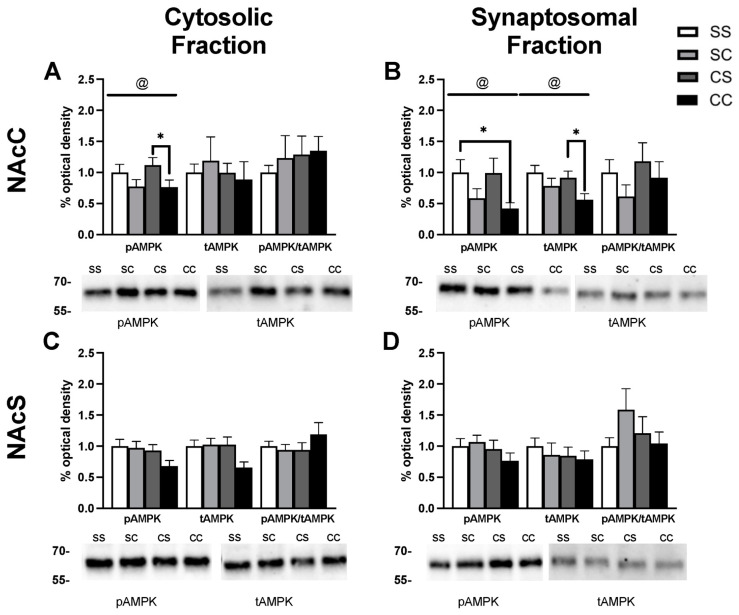
AMPK levels in the NAcC and NAcS of rats in experiment 1. Representative bands and optical densities of pAMPK, tAMPK, and pAMPK/tAMPK in the saline–saline (SS), saline–cocaine (SC), cocaine–saline (CS), and cocaine–cocaine (CC) groups. This order is maintained in the graphs and the bands presented in the representative images. Western band cutouts are from 75 kDa–55 kDa to display pAMPK and tAMPK levels. Protein levels were measured in the cytosolic (left; (**A**,**C**)) and synaptosomal (right; (**B**,**D**)) fractions of the NAcC (top; (**A**,**B**)) and NAcS (right; (**B**,**D**)). Two-way ANOVAs were performed to assess the impact of initial treatment and challenge injections on protein levels. The main effects of challenge injections (@) are denoted in each brain region and subfraction. Sidak’s post hoc tests were performed for differences among treatment groups * *p* < 0.05. (**A**) In the cytosol of the NAcC, there was a main effect of challenge injections on pAMPK (F_1,42_ = 5.780, *p* = 0.0207). (**B**) In the synaptosome of the NACC, there was a main effect of challenge on pAMPK (F_1,42_ = 7.678, *p* = 0.0083) and tAMPK (F_1,35_ = 6.252, *p* = 0.0172). (**C**,**D**) No main effects were found in the cytosol or synaptosome of the NAcS.

In the cytosolic fraction of the NacC, we observed a main effect of challenge injection (F_1,42_ = 5.780, *p* = 0.0207) with rats receiving a cocaine challenge trending toward lower levels of pAMPK (Sidak’s multiple comparisons: *p* = 0.36 for SS vs. SC, *p* = 0.0792 for CS vs. CC) (Figure 3A). In the synaptosomal fraction of the NacC, we similarly observed a main effect of challenge injection for pAMPK (F_1,42_ = 7.678, *p* = 0.0083) and tAMPK (F_1,35_ = 6.252, *p* = 0.0172) with both tending to be reduced following acute cocaine challenge (Figure 3B). There was a decrease in tAMPK in the NAcC synaptosome for repeated cocaine-treated rats compared to repeated saline (*p* = 0.0172). Finally, we failed to find any significant differences in the NAcS (Figure 3C,D), although there was a trend toward an interaction effect for tAMPK levels in the cytosolic fraction (F_1,40_ = 3.604, *p* = 0.0651) and a reduction in tAMPK in the CC group compared to CS (*p* = 0.0426) and SS (*p* = 0.0557) (Figure 3C). Altogether, in this analysis, the most prominently observed treatment effects were associated with cocaine challenge regardless of repeated treatment or highlighted differences between the repeated cocaine-treated groups CS and CC. 

### 2.3. Effects of Metformin Pretreatment on Cocaine-Induced Locomotion and Sensitization

**Figure 4:**
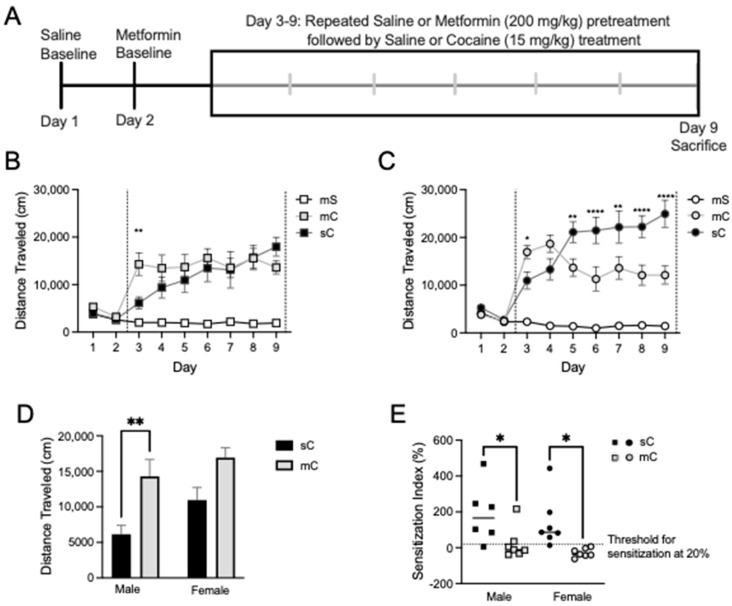
Experiment 2 timeline and behavioral data. (**A**) Timeline of injections administered in experiment 2. (**B**) Distance traveled (cm) by male rats in the metformin + saline (mS), metformin + cocaine (mC), and saline + cocaine (sC) groups on days 1–9. A two-way ANOVA revealed a main effect of treatment F_2,16_ = 13.50, *p* = 0.0004, day F_3.26,51.80_ = 19.92, *p* < 0.0001), and a treatment x day interaction (F_16,127_ = 8.989, *p* < 0.0001). * *p* < 0.05, ** *p* < 0.001 (**C**) Distance traveled (cm) by female rats in groups mS, mC, and sC on days 1–9. A two-way ANOVA revealed a main effect of treatment F_2,16_ = 33.26, *p* < 0.0001, a main effect of day F_3.25,51.11_ = 27.88, *p* < 0.0001, and a treatment x day interaction F_16,126_ = 17.34, *p* < 0.001, **** *p* < 0.0001. (**D**) Distance traveled by males and females in groups sC and mC on day 3. A two-way ANOVA revealed a main effect of treatment group F_1,22_ = 15.52, *p* = 0.0007, and sex F_1,22_ = 4.346, *p* = 0.0489. (**E**) Sensitization index of male and female rats in groups sC and mC. A main effect of treatment was revealed (F_1,23_ = 14.03, *p* = 0.0011).

Rats in experiment two were given injections following the timeline depicted in Figure 4A. Locomotor activity was recorded following each daily injection across three treatment groups: saline + cocaine, metformin + saline, and metformin + cocaine. In this experiment, we incorporated an additional baseline metformin injection on day 2 for all rats. While locomotor activity decreased from day 1 (saline) to day 2 (metformin), this decrease was consistent with normal locomotor habituation. Likewise, daily metformin pretreatment prior to saline injections had no impact on locomotor activity in males or females, with their locomotion resembling that of the negative control saline–saline group in experiment one. We concluded that metformin on its own did not alter locomotor activity. 

Metformin did appear to potentiate the acute stimulatory effects associated with the initial cocaine injection with a two-way ANOVA indicating a main effect of treatment group F_1,22_ = 15.52, *p* = 0.0007, a main effect of sex F_1,22_ = 4.346, *p* = 0.0489, but no interaction F_1,22_ = 0.3714, *p* = 0.5485 (Figure 4D). There was again a requirement for locomotor activity to be increased by ≥20% from the first to the last cocaine injection in the positive control saline + cocaine group, and here one male and one female rat were excluded from the later analysis of protein expression due to failure to reach this threshold (Figure 4E). In males, a two-way mixed-effects ANOVA showed a main effect of treatment F_2,16_ = 13.50, *p* = 0.0004, a main effect of day F_3.26,51.80_ = 19.92, *p* < 0.0001), and a treatment x day interaction (F_16,127_ = 8.989, *p* < 0.0001) (Figure 4B). Dunnett’s post hoc comparisons illustrated within-subjects effects across time indicative of psychomotor sensitization with repeated cocaine administration increasing the distance traveled from day 3 to days 6, 8, and 9 in the saline + cocaine group (*p* = 0.0407, *p* = 0.0143, and =0.0024). In contrast, in the metformin + cocaine group, day 3 locomotion only differed from baseline days 1 and 2 saline and metformin treatment (Dunnett’s post hoc: *p* = 0.02, *p* = 0.0131). In females, two-way mixed-effects ANOVA showed a main effect of treatment F_2,16_ = 33.26, *p* < 0.0001, a main effect of day F_3.25,51.11_ = 27.88, *p* < 0.0001, and a treatment x day interaction F_16,126_ = 17.34, *p* < 0.0001) (Figure 4C). Dunnett’s post hoc comparisons illustrated within-subjects effects across time indicative of psychomotor sensitization with repeated cocaine administration increasing the distance traveled from day 3 to days 6–9 in the saline + cocaine group (*p* = 0.027, *p* = 0.0114, *p* = 0.0041 and *p* = 0.01). In the metformin + cocaine group, day 3 locomotion only differed from baseline days 1 and 2 saline and metformin treatment (Dunnett’s post hoc: *p* = 0.0011 and *p* = 0.0003). When the sensitization index was directly compared between saline and metformin pre-treated subjects, it further illustrated the fact that metformin blocked the development of locomotor sensitization in males (Tukey’s post hoc, *p* = 0.0171) and females (Tukey’s post hoc, *p* = 0.0119) (two-way ANOVA main effect of treatment F_1,23_ = 14.03, *p* = 0.0011) (Figure 4E). These data illustrate the dual effects of metformin to potentiate the acute locomotor stimulating effects of cocaine but disrupt the ability of cocaine to produce sensitization. 

### 2.4. Effects of Metformin Pretreatment Followed by Cocaine on AMPK Protein Levels

**Figure 5:**
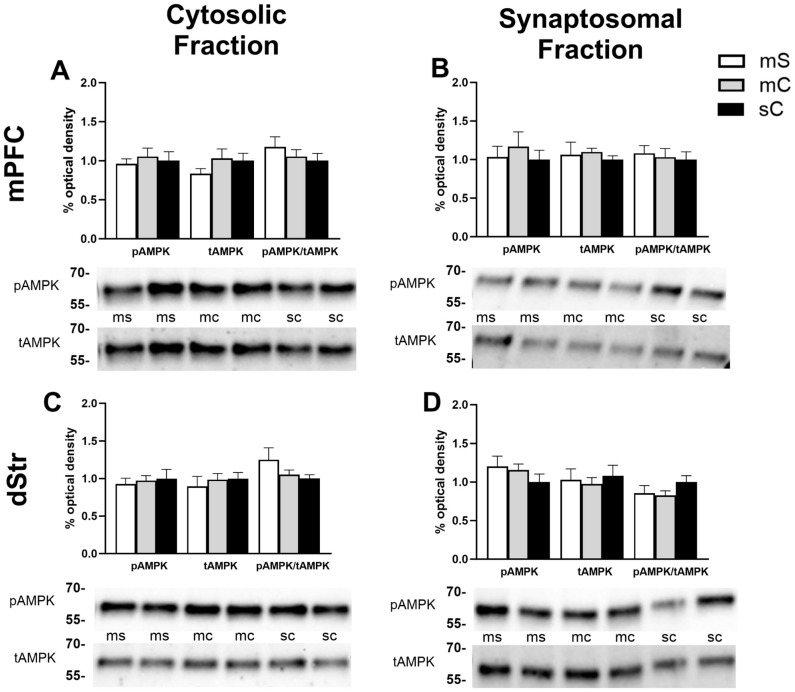
AMPK levels in the mPFC and dStr of rats in experiment 2. Representative bands and optical densities of pAMPK, tAMPK, and pAMPK/tAMPK in the metformin + saline (mS), metformin + cocaine (mC), and saline + cocaine (sC) groups. This order is maintained in the graphs and bands presented in the representative images. Western band cutouts are from 75 kDa–55 kDa to display pAMPK and tAMPK levels. Protein levels were measured in the cytosolic (left; (**A**,**C**)) and synaptosomal (right; (**B**,**D**)) fractions of the mPFC (top; (**A**,**B**)) and dStr (right; (**B**,**D**)). One-way ANOVAs were performed to assess the impact of treatment on protein levels. No effects of treatment were found in the cytosol or synaptosome of the mPFC or dStr.

**Figure 6:**
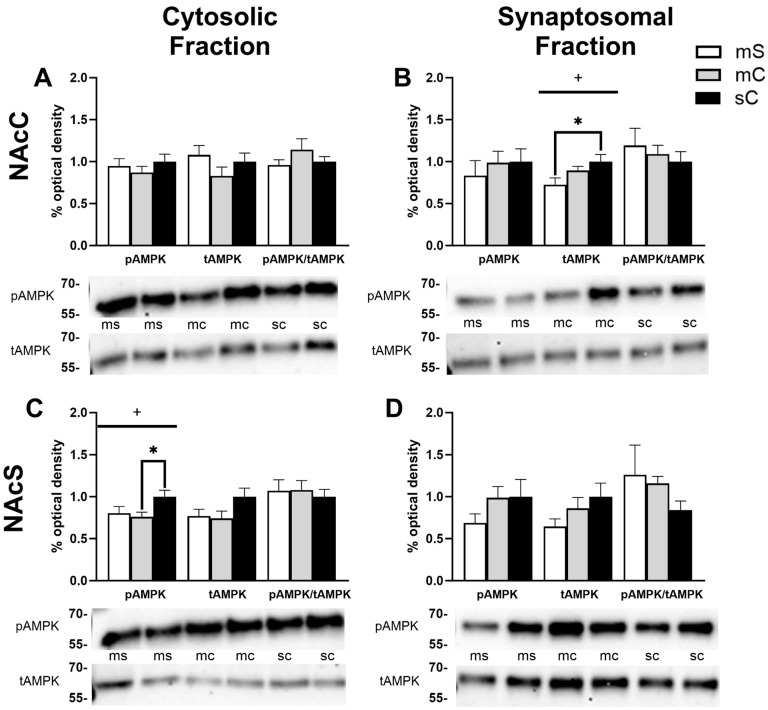
AMPK levels in the NAcC and NAcS of rats in experiment 2. Representative bands and optical densities of pAMPK, tAMPK, and pAMPK/tAMPK in the metformin + saline (mS), metformin + cocaine (mC), and saline + cocaine (sC) groups. This order is maintained in the graphs and bands presented in the representative images. Western band cutouts are from 75 kDa–55 kDa to display pAMPK and tAMPK levels. Protein levels were measured in the cytosolic (left; (**A**,**C**)) and synaptosomal (right; (**B**,**D**)) fractions of the NAcC (top; (**A**,**B**)) and NAcS (right; (**B**,**D**)). One-way ANOVAs were performed to assess the impact of treatment on protein levels. The main effects of treatment (+) are denoted in each brain region and subfraction. Dunnett’s post hoc tests were performed to identify differences among treatment groups. (**A**) Treatment had no effect in the cytosol of the NAcC. (**B**) In the synaptosome of the NAcC, treatment had an effect on tAMPK levels (F_2,33_ = 3.373, *p* = 0.0465). * *p* < 0.05 when comparing tAMPK in the mS and sC groups. (**C**) In the cytosol of the NAcS, treatment had an effect on pAMPK levels (F_2,31_ = 3.353, *p* = 0.0481). * *p* < 0.05 when comparing pAMPK in the mC and sC groups. (**D**) No effect of treatment was seen in the synaptosome of the NAcS.

Given the pronounced effects of metformin on cocaine-induced locomotor responses, we similarly expected to observe large changes in the pAMPK protein levels. To our surprise, we observed limited differences between treatment groups. Indeed, we report no significant differences in the cytosolic or synaptosomal fractions of the mPFC or dStr for pAMPK, tAMPK, or pAMPK/tAMPK (Figure 5). We observed a treatment effect for tAMPK in the synaptosomal fraction of the NAcC (one-way ANOVA F_2,33_ = 3.373, *p* = 0.0465), with an increase in tAMPK for the saline + cocaine group compared to metformin + saline (Dunnett’s post hoc: *p* = 0.0266) (Figure 6B). In the NAcS, there was a treatment effect for pAMPK in the cytosolic fraction (one-way ANOVA F_2,31_ = 3.353, *p* = 0.0481) with paradoxically lower levels of pAMPK in the metformin + cocaine vs. saline + cocaine groups (Dunnett’s post hoc: *p* = 0.0266) (Figure 6C). 

**Figure 7:**
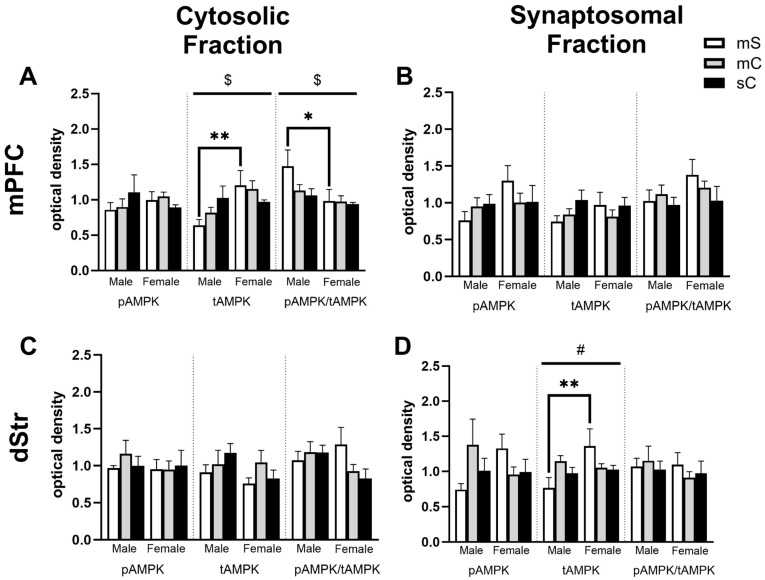
Sex differences in the mPFC and dStr of rats in experiment 2. Treatment groups included metformin + saline (mS), metformin + cocaine (mC), and saline + cocaine (sC). Protein levels were measured in the cytosolic (left; (**A**,**C**)) and synaptosomal (right; (**B**,**D**)) fractions of the mPFC (top; (**A**,**B**)) and dStr (right; (**B**,**D**)). Two-way ANOVAs were performed to assess the impact of treatment and sex on protein levels. There were no main effects of chronic treatment found. Main effects of sex ($) and the interaction between chronic and challenge injections (#) are denoted in each brain region and subfraction. Sidak’s post hoc tests were performed to determine specific sex differences in treatment groups, with * *p* < 0.05, ** *p* < 0.01. (**A**) A main effect of sex was seen in tAMPK (F_1,31_ = 8.214, *p* = 0.0074) and pAMPK/tAMPK (F_1,30_ = 5.389, *p* = 0.0272) levels in the mPFC’s cytosol. (**B**,**C**) No main effects of sex were seen. (**D**) In the synaptosome of the dStr, a main effect of sex was seen for tAMPK levels (F_1,27_ = 4.874, *p* = 0.0359).

**Figure 8:**
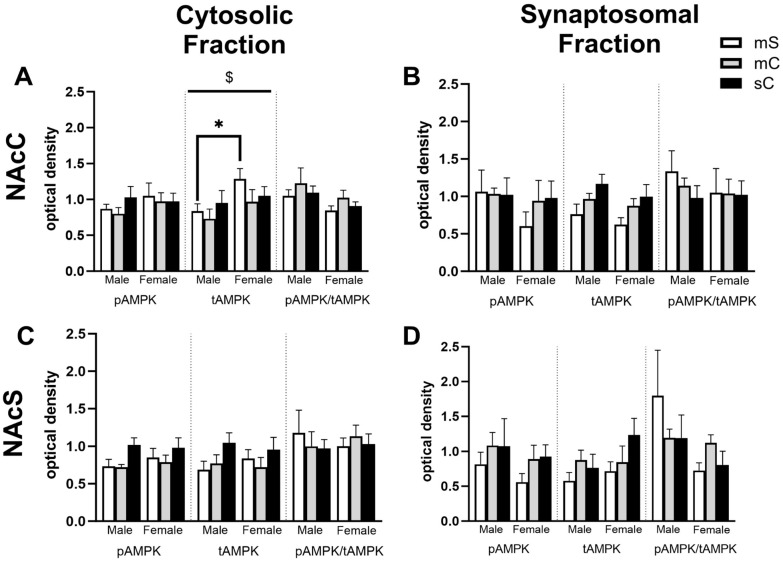
Sex differences in the NAcC and NAcS of rats in experiment 2. Treatment groups were metformin + saline (mS), metformin + cocaine (mC), and saline + cocaine (sC). Protein levels were measured in the cytosolic (left; (**A**,**C**)) and synaptosomal (right; (**B**,**D**)) fractions of the NAcC (top; (**A**,**B**)) and NAcS (right; (**B**,**D**)). Two-way ANOVAs were performed to assess the impact of treatment and sex on protein levels. There were no main effects of chronic treatment found. Main effects of sex ($) are denoted in each brain region and subfraction. Sidak’s post hoc tests were performed to determine specific sex differences in the treatment groups, with * *p* < 0.05. (**A**) In the cytosol of the NAcC, there was a main effect of sex on tAMPK levels (F_1,27_ = 4.874, *p* = 0.0359). (**B**–**D**) No main effects of sex were observed.

Upon closer examination of the data, it was found that in some cases a lack of overall treatment effect was observed due to sex differences, and this experiment was sufficiently powered to further examine these effects. For example, with respect to tAMPK in the cytosolic fraction of the mPFC, when analyzed by two-way ANOVA we found a main effect of sex (F_1,31_ = 8.214, *p* = 0.0074), no main effect of treatment (F_2,31_ = 0.2182, *p* = 0.8052), and almost a sex x treatment interaction (F_2,31_ = 3.228, *p* = 0.0533) (Figure 7A). In female rats compared to male rats, tAMPK was higher in the metformin + saline group (*p* = 0.0109). There was a similar story for pAMPK/tAMPK with a two-way ANOVA indicating a main effect of sex (F_1,30_ = 5.389, *p* = 0.0272), but in this case, pAMPK/tAMPK was lower in female rats compared to male rats (Sidak’s post-test, *p* = 0.0469) (Figure 7A). In the synaptosomal fraction of the dStr, there was a trend for an interaction between sex and treatment for levels of pAMPK (F_2,32_ = 2.776, *p* = 0.0773) and a significant interaction but no main effects for tAMPK (F_2,32_ = 4.096, *p* = 0.0261) (Figure 7D). Levels of tAMPK were higher in metformin + saline females compared to males (*p* = 0.0081). In the cytosolic fraction of NAcC, there was a main effect of sex for tAMPK levels (F_1,27_ = 4.874, *p* = 0.0359) (Figure 8A). Levels of tAMPK were again higher in metformin + saline females compared to males (*p* = 0.0379). These data suggest sex differences in the molecular effects of metformin in the absence of cocaine in some brain regions, which may have obscured other treatment group effects. Moreover, pAMPK levels, predicted to be increased by metformin, were only changed in the NAcS in the direction opposite to our expectations (Figure 6C). 

## 3. Discussion

In this study, we used cocaine locomotor sensitization to understand the role of AMPK in mediating the effects of cocaine. The first experiment provided confirmatory results relating to sex differences in the locomotor response to cocaine [42]. We found that while the acute locomotor response to cocaine was larger in female rats, repeated cocaine injections tended to produce greater psychomotor sensitization in male rats. We examined the levels of phosphorylated and total AMPK in cytosolic and synaptosomal fractions of lysates from multiple brain regions in the corticostriatal circuit. The analysis was conducted across four treatment groups (SS, SC, CS, and CC) to probe the acute and repeated cocaine effects. The most prominently observed treatment effects were associated with cocaine challenge regardless of repeated treatment with saline or cocaine. Most frequently, we detected differences post hoc between the repeated cocaine-treated groups CS and CC.

In the second experiment, we tested the effects of metformin, an indirect AMPK activator, on cocaine sensitization. We identified dual effects of metformin with augmentation of the acute effects of cocaine but a disruption in sensitization. Contrary to our predictions, we observed a decrease rather than an increase in pAMPK in the NAcS associated with metformin pretreatment prior to cocaine. Despite large behavioral effects, overall treatment group effects were less robust, in some cases due to sex differences or sex by treatment interactions. The lack of consistent regulation of AMPK may suggest a role for an alternative mediator of the effects of metformin. 

AMPK is mostly cytosolic, but it can also localize to cellular membranes [41], and dynamic protein interactions at or near synapses are known to be important for the plasticity associated with behavioral sensitization [43]. Cocaine sensitization had significant impacts on total protein and phosphorylation levels of AMPK in various regions of the corticostriatal reward circuit. We predicted that cocaine sensitization would be associated with increased pAMPK in the mPFC and decreased pAMPK in the dorsal and ventral striatum (dStr, NAcC, and NAcS). This prediction was based on prior studies that had examined AMPK levels in whole cell lysates and observed dose-dependent increases or decreases in pAMPK/tAMPK expression in the mPFC and dStr, respectively [26]. Our work expands upon that study, filling a gap in the literature by examining additional ventral striatum brain regions, separately querying the cytosolic and synaptosomal fractions, and including both male and female rats. Moreover, we also examined the levels of total and phosphorylated AMPK separately given that intracellular trafficking could impact both independently. In our studies, cocaine challenge was associated with an increase in pAMPK/tAMPK in the cytosol of the mPFC. In the synaptosome, there was an interaction between acute and repeated treatment effects for pAMPK, such that the largest difference emerged between the CC and CS groups. This overall result is only somewhat consistent with the prior study demonstrating increased pAMPK/tAMPK in whole cell lysates from mPFC following acute and repeated cocaine treatment in male rats [26]. In the dStr, our results in cytosolic and synaptosomal fractions were not consistent with a dose-dependent decrease in pAMPK/tAMPK associated with cocaine treatment, as found by Xu and Kang. In the NAcC, cocaine challenge was associated with decreased pAMPK in the cytosol and synaptosome and decreased tAMPK in the synaptosome. No changes were observed in the NAcS. There could be a number of explanations for the disparate findings between these studies, including differences in dissections, the inclusion of female rats, and, of course, the examination of subcellular fractions compared to whole cell lysates. Unfortunately, our ability to make more direct comparisons is limited because we do not have total protein fractions from our experimental animals. 

Spatially and temporally dynamic AMPK activity allows for distinct signaling in subcellular compartments for precise control of cellular functions [44]. We observed differences in pAMPK, tAMPK, and pAMPK/tAMPK in cytosolic and synaptosomal subcellular compartments of different brain regions. AMPK also shuttles between the nucleus and the cytoplasm, with AMPK ɑ2 containing both a nuclear localization signal sequence (NLS) and a nuclear export signal sequence (NES) [45], although in this study, we did not examine protein changes in the nuclear fraction. A number of stimuli are known to impact AMPK nuclear–cytoplasmic shuttling, including starvation, heat shock, oxidative stress, and circadian signals, but what regulates the localization of AMPK to the synaptosome is less well-established [41]. It has been shown that, at least in cortical tissues, the kinase activity of AMPK shows enrichment in both nuclear and synaptosomal fractions. The detection of significant differences in tAMPK in our experiments suggests that we are capturing some level of AMPK trafficking associated with cocaine treatment. This dynamic trafficking may also contribute to why we failed to replicate the dose-dependent effects on pAMPK previously observed with whole cell lysates. 

The most intriguing result of this study was the finding that metformin pretreatment 30 min prior to each cocaine injection prevented the induction of cocaine sensitization. While their overall locomotion levels were still high compared to rats that had only received metformin + saline, the increase in locomotion across days that would indicate sensitization was absent. In female rats, there was a trend of locomotion even decreasing after the first few exposures to cocaine. Metformin on its own did not seem to have an impact on activity in line with previous studies [46], although metformin treatment can improve locomotor coordination and balance in control mice and in the context of spinal cord injury or neurodegeneration [47,48,49]. Surprisingly, metformin pretreatment increased the acute locomotor response to cocaine. We are conducting studies to determine if there are any differences in the impact of acute and prolonged metformin and cocaine exposure on the expression of AMPK or other molecular markers associated with cocaine-induced plasticity. It is worth noting that all rats in our second experiments had prior exposure to metformin due to their day 2 baseline injection. However, based on their subsequent observed behavior, we have no reason to believe that this acute metformin injection had any long-term impact on any of the treatment groups.

Protein analysis revealed few differences between treatment groups in the metformin experiment, with none in the mPFC and dStr. This may partially be explained due to the absence of a short-term withdrawal group in this experiment, given that many of the observed differences in experiment 1 were between the CS and CC groups. Future studies will further interrogate the impact of acute or protracted withdrawal on AMPK activation and trafficking following cocaine sensitization and in conjunction with metformin treatment. Another reason for the lack of overall treatment group effects in this experiment was the apparent sex differences, especially for tAMPK levels, between rats receiving daily metformin treatment (mS). Metformin is expected to increase AMPK activity (i.e., increase pAMPK), but it may also differentially impact the trafficking of tAMPK between the cytosol and the synapse in male and female rats. Indeed, we were surprised to observe minimal changes in pAMPK levels between the saline + cocaine and metformin + cocaine groups, with only an unexpected decrease in pAMPK noted in the cytosol of the NAc shell associated with metformin pretreatment. As in the first experiment, a potential explanation could be related to our examination of cellular subcompartments instead of whole cell lysates. Alternatively, it may be possible that the observed effect of metformin on cocaine sensitization is mediated through a protein other than AMPK. There are numerous studies in multiple contexts highlighting effects of metformin that may not be solely mediated by AMPK [29]. For example, metformin can inhibit mTOR through an AMPK-independent mechanism by increasing REDD1 expression, which is regulated by p53 and has implications for cancer therapy [50]. Likewise, metformin-mediated regulation of NF-κB inflammatory signaling in primary hepatocytes was unaffected by AMPKɑ knockout [51]. Future work will parse the AMPK-dependent versus AMPK-independent effects of metformin on cocaine-induced locomotor sensitization.

## 4. Materials and Methods

### 4.1. Subjects

Male (*n* = 48) and female (*n* = 54) Sprague-Dawley rats between 8–10 weeks of age were purchased from Envigo/Inotiv (West Lafayette, IN, USA). The male rats weighed ~310–340 grams, while the females were ~210–230 grams. The rats were pair-housed in individually ventilated cages in a Research Animal Resources (RAR) managed facility and kept on a 14:10 light:dark cycle with lights on at 06:00 and lights off at 20:00. Standard chow and water were available *ad libitum*. The room temperature was kept at ~70 °F with a humidity between 38–46%. All animal procedures were performed at the University of Minnesota in accordance with protocols pre-approved by our Institutional Animal Care and Use Committee (IACUC).

### 4.2. Chemicals

Cocaine hydrochloride and euthasol (pentobarbital sodium and phenytoin sodium) were obtained from Boynton Pharmacy (University of Minnesota, Minneapolis, MN, USA). Metformin hydrochloride (B1970) was obtained from ApexBio (Houston, TX, USA). Cocaine and metformin were prepared in sterile 0.9% saline.

### 4.3. Cocaine Sensitization

The animals were delivered to our facility 3–5 days before experiment initiation and handled 3 days prior to beginning behavioral testing. All rats in both experiments were given a baseline intraperitoneal (IP) injection of saline on experimental day one. Locomotion was measured using open-field locomotor arenas fitted with multiple infrared sensors along each side (MedAssociates; St. Albans, VT, USA). Sensors measured the movement of the animals by tracking beam breaks in the x, y, and z directions. Rat locomotion was analyzed to determine if sensitization occurred in rats receiving cocaine injections over multiple days [26]. Rats were considered to have been sensitized if their locomotion increased by at least 20%. 

#### 4.3.1. Experiment 1

**Figure 9:**
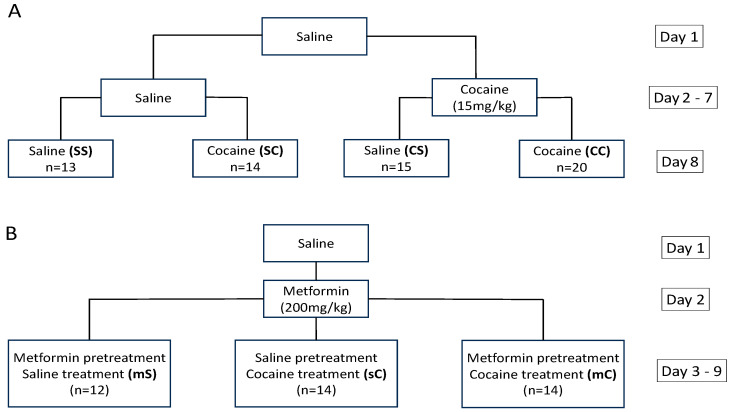
Experimental groups in experiments 1 and 2. (**A**) Baseline, initial, and challenge injections administered to groups in experiment one. The treatment groups were: saline–saline (SS), saline–cocaine (SC), cocaine–saline (CS), and cocaine–cocaine (CC). (**B**) Baseline, pretreatment, and treatment injections administered to groups in experiment two. The treatment groups were: metformin–saline (mS), metformin–cocaine (mC), and saline–cocaine (sC).

Rats were divided into two groups and given IP injections of saline or cocaine (15 mg/kg) for six days (days 2–7). The cocaine dosage was chosen based on similar studies that have shown that it induces strong sensitization within five days [26]. On the eighth day, rats were given a final challenge injection of saline or cocaine such that by the end of the experiment, there were four groups of rats: saline–saline (SS), saline–cocaine (SC), cocaine–saline (CS), and cocaine–cocaine (CC) (Figure 9A). Locomotion was tracked immediately after each injection. The sensitization index for group CC was assessed by comparing locomotion on day 8 to that of day 2. For group CS, locomotion on day 7 was compared to day 2 to determine sensitization effects. The rats were euthanized for tissue collection immediately after recording their locomotion on day 8. 

#### 4.3.2. Experiment 2

The rats were given a baseline injection of saline on day 1 and metformin (IP, 200 mg/kg) on day 2, with locomotion tracked 30 minutes post-injection for 45 minutes on both days. On days 3–9, the rats were split into three groups. Each group was given a pretreatment injection of metformin or saline followed by a treatment injection of saline or cocaine with a thirty-minute inter-injection interval: metformin + saline (mS), metformin + cocaine (mC), or saline + cocaine (sC) (Figure 9B). Following the treatment injection, locomotion was immediately tracked for 45 minutes. The sensitization index was determined by comparing locomotion on day 9 to day 3 for all groups. The rats were euthanized after recording their locomotion on day 9.

### 4.4. Euthanasia, Tissue Extraction, Preparation, and Western Blotting

**Figure 10:**
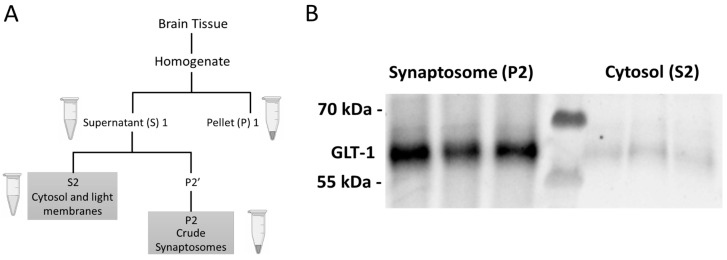
Process and verification of crude fractionation. (**A**) Supernatant (S) and Pellet (P) subfractions obtained from brain tissue through a crude fractionation protocol. (**B**) Western blot image of glutamate transporter 1 imaged in the cytosolic and synaptosomal fractions of the dStr.

Immediately after recording locomotion on the last day, the rats were euthanized with euthasol solution (≥86 mg/kg, IP) prior to decapitation and brain extraction. The mPFC, NAc core (NAcC), NAc shell (NAcS), and dStr were removed and the tissues were immediately frozen on dry ice [52]. Tissue samples were stored at −80° C until further processing. Crude fractionation was performed to obtain specific subcellular compartments of the brain tissue in each region (Figure 10A). Samples were homogenized in 10 MM HEPES/0.32 M sucrose buffer with a pH of 7.4, as previously described [53]. Inhibitors were added to prevent the activation of proteases and phosphatases (78447, ThermoFisher; Waltham, MA, USA). The cytosolic (S2) and the synaptosomal (P2) fractions were extracted to differentially assess protein expression in the cytosolic versus post-synaptic density enriched synaptosome fractions [53]. Protein concentration was determined with the Pierce Rapid Gold BCA assay (A53225, ThermoFisher; Waltham, MA, USA). Western blots were performed as described previously [36]. In brief, electrophoresis was performed according to standard protocols using 4–12% Criterion XT Bis-Tris precast gels (Bio-Rad; Hercules, CA, USA) run in XT-MOPS buffer (Bio-Rad; Hercules, CA, USA) with 10–15 ug total protein loaded per well. Gels were transferred to nitrocellulose membranes using the Trans-Blot Turbo system (Bio-Rad; Hercules, CA, USA). Blocking was performed with fish gelatin (22010, Biotium; Fremont, CA, USA) in Tris-buffered saline (TBS) with 0.1% Tween 20. The primary antibodies were: anti-phospho-AMPKɑ Thr172 (Cell Signaling 2535, 1:500; Danvers, MA, USA), anti-AMPKɑ (Cell Signaling 2532, 1:1000; Danvers, MA, USA), and anti-GAPDH (Cell Signaling 2118, 1:5000; Danvers, MA, USA). HRP-conjugated secondary antibodies (Cell Signaling 7074; Danvers, MA, USA) and Super Signal West Dura ECL reagent (ThermoFisher; Waltham, MA, USA) were used for chemiluminescent detection. Stripping buffer with a pH of 2.2 was made using glycine (Sigma-Aldrich; Burlington, MA, USA) and sodium dodecyl sulfate (SDS) (Fiscer Bioreagents; Waltham, MA, USA). Blots were imaged on an iBright FL1000 (Invitrogen; Waltham, MA, USA), with images analyzed using the integrated analysis software (version 1.8.0). To validate the crude fractionation procedure, we probed for glutamate transporter 1 (GLT1; MilliporeSigma AB1783, 1:500; Burlington, MA, USA). Since GLT1 is primarily localized to the membranes of astrocytic processes near glutamatergic synapses [54], it should be enriched in the P2 but not the S2 fraction [55] (Figure 10B).

### 4.5. Statistical Analysis

Two-way repeated measures analysis of variance (ANOVA) was used to assess changes in locomotor activity across treatment days. The criterion for sensitization was an increase in locomotion of at least 20% between the last and first cocaine injection for the CC and CS groups. An exclusion criterion of 30% was applied to the acute cocaine response for the SC group. For experiment 1, two-way ANOVAs were performed to determine the impact of initial and challenge injections on various protein levels in the regions of interest. To combine data across membranes due to the large number of samples, the optical density of the western blot bands was first normalized within-membrane based on the control band density and then combined with samples probed on other membranes. For experiment 2, one-way ANOVAs were performed to compare the impact of treatments on protein levels in different groups. A mix of male and female samples were run on each gel in order to allow comparisons between sexes. Two-way ANOVAs were performed to determine the impact of sex, treatment, and the interaction of both factors on protein levels. Data are presented as the mean ± standard error of the mean (SEM). Analyses with *p* < 0.05, with post hoc testing as appropriate, were deemed statistically significant. The ROUT test was run with Q = 0.5% to identify potential outliers, but samples were only excluded based on the appearance of the western blot image (e.g., excessive background, incomplete bands). Complete information about the statistical analysis is given in Supplemental Table S1. GraphPad PRISM (version 10) was used to analyze the results of the experiments.

## 5. Conclusions

In conclusion, this study explored the interplay between cocaine-induced locomotor sensitization and AMPK activation and trafficking in corticostriatal reward circuitry. Surprisingly, rather than observing graded dose-response-like effects, the most frequently observed treatment effects were associated with cocaine challenge irrespective of prior repeated treatment. This protein analysis revealed the nuanced dynamics of AMPK and pAMPK in distinct brain regions and subcellular compartments in response to cocaine. This study is not without limitations. For example, while these results suggest dynamic trafficking of phosphorylated and total AMPK, we were unable to verify this by examining total protein fractions. Similarly, we did not interrogate the expression of these proteins in the nuclear fraction, where AMPK is known to be involved in the regulation of gene expression [56,57]. This function could be important for some of the cocaine-induced adaptations associated with sensitization [58]. 

Our second experiment provides insights into the complex responses associated with repeated cocaine exposure and metformin pretreatment. Notably, metformin produced an unexpected increase in the acute locomotor response to cocaine, but importantly, it still prevented the development of cocaine sensitization. Our sensitization protocol was based on prior studies [26], but only a single cocaine dose and a single metformin dose were tested in those experiments. Given the observed functional interaction between these drugs, future studies should explore additional dose-response relationships between metformin and cocaine. At the molecular level, the observed sex differences, especially in tAMPK levels, underscore the importance of considering sex-related variations in future investigations. Finally, as we continue to elucidate the metformin-cocaine interaction, we must acknowledge that metformin’s effects may extend beyond the traditional AMPK pathway examined here, presenting an exciting avenue for future research.

## Data Availability

The data presented in this study are available on request from the corresponding author.

## Supplementary Materials

The supporting information can be downloaded at: https://www.mdpi.com/article/10.3390/ijms242316859/s1.
