# Supplementary material for: Metformin Prevents Cocaine Sensitization: Involvement of Adenosine Monophosphate-Activated Protein Kinase Trafficking between Subcellular Compartments in the Corticostriatal Reward Circuit
Source: Int J Mol Sci. 2023 Nov 28;24(23):16859. doi: 10.3390/ijms242316859 (PMC10706784; doi:10.3390/ijms242316859)
Supplement: Supplementary file 1 [file ijms-24-16859-s001.zip › ijms-2707933-supplementary.pdf]

Supplementary Figures and Tables:

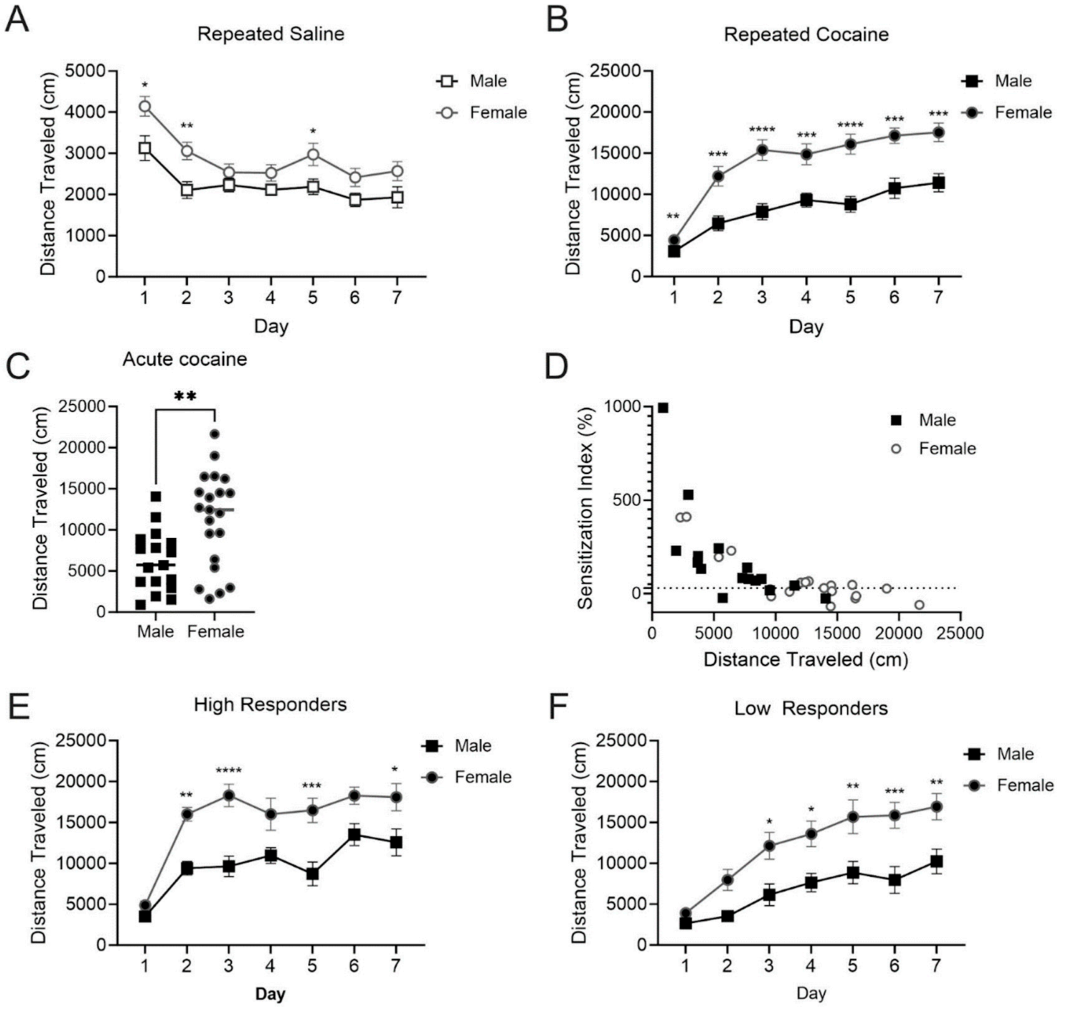

Figure S1. Sex differences in cocaine locomotor sensitization. A) Distance travelled by all male and female rats exposed to saline as an initial treatment over the course of experiment 1. A two-way ANOVA revealed a main effect of day $F_{3.57,89.28}=30.48$, $p<0.0001$, sex $F_{1,25}=6.255$, $p=0.0193$, and a sex x day interaction $F_{6,150}=2.162$, $p=0.0498$. *p<0.05, **p<0.01. **B)** Distance travelled by all male and female rats exposed to 15 mg/kg of cocaine as an initial treatment over the course of experiment 1. A main effect of day $F_{3.64,120.2}=30.95$, $p<0.0001$, a sex $F_{1,33}=46.2$, $p<0.0001$, and a sex x day interaction $F_{6,198}=2.478$, $p=0.0247$. ***p<0.001, ****p<0.0001 **C)** Distance travelled by all male and female rats after acute exposure to 15 mg/kg of cocaine 1. A Mann Whitney Test revealed a significant difference in response to acute cocaine, p=0.0048. **D)** Relationship between sensitization index and distance travelled by male ($R^2=0.4615$, $p=0.0038$) and female ($R^2=0.7043$, $p<0.0001$) rats after acute exposure to cocaine**. E)** Distance travelled by rats deemed high responders to cocaine over the course of the initial treatment injections. The results of a two-way ANOVA revealed a main effect of day $F_{6,112}=17.86$, $p<0.0001$, sex $F_{1,112}=65.98$, $p<0.0001$, and a group x day interaction $F_{18,186}=2.265$, $p=0.0034$. **F)** Distance travelled by rats deemed low responders to cocaine over the course of the initial treatment injections. A main effect of day $F_{6,105}=14.45$, $p<0.0001$ and sex $F_{1,105}=55.88$, $p<0.0001$ were found.

Table S1. Statistical analysis

**Figure number**	**Test**	**F-value/U-value**	**P-value**	**Partial Eta^2**
Figure 3B	Mann-Whitney test, two-tailed	U=88	0.0343	
Figure 3C	Two-way ANOVA<br>Treatment<br>Sex<br>Treatment x Sex	 F(1,34) = 14.46<br>F(1,34) = 59.05<br>F(1,34) = 10.14	 p<0.0001<br>p=0.0006<br>p=0.0031	 0.634<br>0.372<br>0.230
Figure 3D	RM Two-way ANOVA<br>Treatment<br>Day<br>Treatment x Day	 F(3,20) = 22.81<br>F(3.274, 74.49) = 9.419<br>F(21,140) = 4.774	 p<0.0001<br>p<0.0001<br>p<0.0001	 0.700<br>0.320<br>0.505
Figure 3E	RM Two-way ANOVA<br>Treatment<br>Day<br>Treatment x Day	 F(3,21) = 56.70<br>F(3.677, 77.22) = 13.35<br>F(21,147) = 4.072	 p<0.0001<br>p<0.0001<br>p<0.0001	 0.825<br>0.389<br>0.675
Figure 4A<br>pAMPK<br><br><br>tAMPK<br><br><br>pAMPK/tAMPK	Two-way ANOVA<br>Repeated Injection<br>Acute Injection<br>Interaction<br>Repeated Injection<br>Acute Injection<br>Interaction<br>Repeated Injection<br>Acute Injection<br>Interaction	 F(1,37) = 0.5551<br>F(1,37) = 0.00116<br>F(1,37) = 0.2835<br>F(1,41) = 0.6737<br>F(1,41) = 0.2508<br>F(1,37) = 0.05196<br>F(1,37) = 1.130<br>F(1,37) = 6.580<br>F(1,37) = 0.01567	 p=0.4609<br>p=0.9730<br>p=0.5976<br>p=0.4165<br>p=0.6192<br>p=0.8208<br>p=0.2948<br>p=0.0145<br>p=0.9011	 0.0148<br>0.0000313<br>0.0076<br>0.0162<br>0.00608<br>0.0013<br>0.0296<br>0.1508<br>0.000423
Figure 4B<br>pAMPK<br><br><br>tAMPK<br><br><br>pAMPK/tAMPK	Two-way ANOVA<br>Repeated Injection<br>Acute Injection<br>Interaction<br>Repeated Injection<br>Acute Injection<br>Interaction<br>Repeated Injection<br>Acute Injection<br>Interaction	 F(1,41) = 0.6871<br>F(1,41) = 1.690<br>F(1,41) = 5.070<br>F(1,29) = 3.140<br>F(1,29) = 1.285<br>F(1,29) = 0.5119<br>F(1,29) = 0.04568<br>F(1,29) = 1.702<br>F(1,29) = 0.2289	 p=0.4119<br>p=0.2009<br>p=0.0298<br>p=0.0869<br>p=0.2663<br>p=0.4800<br>p=0.8323<br>p=0.2023<br>p=0.6359	 0.0165<br>0.0396<br>0.1100<br>0.0977<br>0.0424<br>0.0173<br>0.0016<br>0.0555<br>0.0078

Figure 4C	Two-way ANOVA			
pAMPK	Repeated Injection	F(1,41) = 0.1575	p=0.6935	0.0038
	Acute Injection	F(1,41) = 3.509	**p=0.0682**	**0.0788**
	Interaction	F(1,41) = 0.4904	p=0.4877	0.0118
tAMPK	Repeated Injection	F(1,40) = 0.2308	p=0.6336	0.0057
	Acute Injection	F(1,40) = 0.3858	p=0.5380	0.0096
	Interaction	F(1,40) = 5.947	p=0.0193	0.1294
pAMPK/tAMPK	Repeated Injection	F(1,40) = 0.2036	p=0.6543	0.0051
	Acute Injection	F(1,40) = 2.357	p=0.1326	0.0556
	Interaction	F(1,40) = 5.106	p=0.0294	0.1132
Figure 4D	Two-way ANOVA			
pAMPK	Repeated Injection	F(1,41) = 0.9455	p=0.3366	0.0225
	Acute Injection	F(1,41) = 0.4618	p=0.5006	0.0111
	Interaction	F(1,41) = 0.002878	p=0.9576	0.000070
tAMPK	Repeated Injection	F(1,40) = 0.1673	p=0.6847	0.0042
	Acute Injection	F(1,40) = 1.382	p=0.2467	0.0334
	Interaction	F(1,40) = 1.097	p=0.3012	0.0267
pAMPK/tAMPK	Repeated Injection	F(1,39) = 2.160	p=0.1497	0.0525
	Acute Injection	F(1,39) = 0.05351	p=0.8183	0.0014
	Interaction	F(1,39) = 1.586	p=0.2154	0.0391
Figure 5A	Two-way ANOVA			
pAMPK	Repeated Injection	F(1,42) = 0.2089	p=0.6500	0.0049
	Acute Injection	F(1,42) = 5.780	p=0.0207	0.0121
	Interaction	F(1,42) = 0.2770	p=0.6015	0.0066
tAMPK	Repeated Injection	F(1,40) = 0.339	p=0.5637	0.0084
	Acute Injection	F(1,40) = 0.02257	p=0.8813	0.00056
	Interaction	F(1,40) = 0.3071	p=0.5825	0.0076
pAMPK/tAMPK	Repeated Injection	F(1,39) = 0.5589	p=0.4592	0.0141
	Acute Injection	F(1,39) = 0.2996	p=0.5873	0.0076
	Interaction	F(1,39) = 0.09976	p=0.7538	0.0026
Figure 5B	Two-way ANOVA			
pAMPK	Repeated Injection	F(1,42) = 0.2300	p=0.6340	0.0054
	Acute Injection	F(1,42) = 7.687	p=0.0083	0.1545
	Interaction	F(1,42) = 0.1776	p=0.6756	0.0042
tAMPK	Repeated Injection	F(1,35) = 1.804	p=0.1879	0.049
	Acute Injection	F(1,35) = 6.252	p=0.0172	0.1515
	Interaction	F(1,35) = 0.3359	p=0.5659	0.0095
pAMPK/tAMPK	Repeated Injection	F(1,34) = 0.9479	p=0.3317	0.0271
	Acute Injection	F(1,34) = 1.749	p=0.1949	0.0489
	Interaction	F(1,34) = 0.05753	p=0.8119	0.0017
Figure 5C	Two-way ANOVA			
pAMPK	Repeated Injection	F(1,39) = 3.286	**p=0.0776**	**0.0777**

	Acute Injection	F(1,39) = 1.966	p=0.1688	0.0479
	Interaction	F(1,39) = 1.241	p=0.2722	0.0308
tAMPK	Repeated Injection	F(1,39) = 2.788	p=0.1030	0.0667
	Acute Injection	F(1,39) = 2.766	p=0.1043	0.06620
	Interaction	F(1,39) = 3.604	**p=0.0681**	**0.0846**
pAMPK/tAMPK	Repeated Injection	F(1,39) = 0.5414	p=0.4663	0.0137
	Acute Injection	F(1,39) = 0.5094	p=0.4769	0.0129
	Interaction	F(1,39) = 1.456	p=0.2348	0.0360
Figure 5D	Two-way ANOVA			
pAMPK	Repeated Injection	F(1,42) = 1.904	p=0.1749	0.0434
	Acute Injection	F(1,42) = 0.239	p=0.6275	0.0057
	Interaction	F(1,42) = 1.021	p=0.3182	0.0237
tAMPK	Repeated Injection	F(1,36) = 0.5738	p=0.4537	0.0157
	Acute Injection	F(1,36) = 0.4839	p=0.4911	0.0133
	Interaction	F(1,36) = 0.07614	p=0.7842	0.0021
pAMPK/tAMPK	Repeated Injection	F(1,36) = 0.5156	p=0.4774	0.0141
	Acute Injection	F(1,36) = 0.8092	p=0.3743	0.0220
	Interaction	F(1,36) = 2.623	p=0.1140	0.0678
Figure 6B	Mixed-effects ANOVA			
	Treatment	F(2,16) = 13.50	p<0.0001	
	Day	F(3.263,51.80) = 19.92	p=0.0004	
	Treatment x Day	F(16,127) = 8.989	p<0.0001	
Figure 6C	Mixed-effects ANOVA			
	Treatment	F(2,16) = 33.26	p<0.0001	
	Day	F(3.245,51.11) = 27.88	p<0.0001	
	Treatment x Day	F(16,126) = 17.34	p<0.0001	
Figure 6D	Two-way ANOVA			
	Treatment	F(1,22) = 12.52	p=0.0007	0.414
	Sex	F(1,22) = 4.346	p=0.0489	0.165
	Treatment x Sex	F(1,22) = 0.3714	p=0.5485	0.017
Figure 6E	Two-way ANOVA			
	Treatment	F(1,23) = 14.03	p=0.0011	0.3789
	Sex	F(1,23) = 1.219	p=0.2809	0.0503
	Treatment x Sex	F(1,23) = 0.9697	p=0.9697	0.000064
Figure 7A	One-way ANOVA			
	pAMPK	F(2,35) = 0.1595	p=0.8532	0.0090
	tAMPK	F(2,34) = 0.3029	p=0.7403	0.0175
	pAMPK/tAMPK	F(2,34) = 0.1790	p=0.1824	0.0953

Figure 7B	One-way ANOVA<br>pAMPK<br>tAMPK<br>pAMPK/tAMPK	 F(2,34) = 0.1923<br>F(2,34) = 1.337<br>F(2,34) = 0.9907	 p=0.8260<br>p=0.2762<br>p=0.3818	 0.0112<br>0.0729<br>0.0551
Figure 7C	One-way ANOVA<br>pAMPK<br>tAMPK<br>pAMPK/tAMPK	 F(2,35) = 0.2295<br>F(2,35) = 1.121<br>F(2,35) = 0.7364	 p=0.7961<br>p=0.3373<br>p=0.4861	 0.0129<br>0.0602<br>0.0404
Figure 7D	One-way ANOVA<br>pAMPK<br>tAMPK<br>pAMPK/tAMPK	 F(2,35) = 0.3209<br>F(2,35) = 0.2616<br>F(2,35) = 0.1448	 p=0.7276<br>p=0.7713<br>p=0.8657	 0.0180<br>0.0147<br>0.0082
Figure 8A	One-way ANOVA<br>pAMPK<br>tAMPK<br>pAMPK/tAMPK	 F(2,28) = 0.6681<br>F(2,30) = 1.485<br>F(2,28) = 0.9776	 p=0.5207<br>p=0.2427<br>p=0.3887	 0.0455<br>0.0901<br>0.0653
Figure 8B	One-way ANOVA<br>pAMPK<br>tAMPK<br>pAMPK/tAMPK	 F(2,33) = 0.3278<br>F(2,33) = 3.373<br>F(2,33) = 0.4295	 p=0.7228<br>p=0.0465<br>p=0.6544	 0.0195<br>0.1697<br>0.0254
Figure 8C	One-way ANOVA<br>pAMPK<br>tAMPK<br>pAMPK/tAMPK	 F(2,31) = 3.353<br>F(2,32) = 2.456<br>F(2,31) = 0.1661	 p=0.0481<br>p=0.1018<br>p=0.8477	 0.1778<br>0.1331<br>0.0106
Figure 8D	One-way ANOVA<br>pAMPK<br>tAMPK<br>pAMPK/tAMPK	 F(2,35) = 1.295<br>F(2,35) = 1.738<br>F(2,34) = 0.9889	 p=0.2868<br>p=0.1907<br>p=0.3827	 0.0689<br>0.0904<br>0.0549
Figure 9A<br>pAMPK<br><br><br>tAMPK<br><br><br>pAMPK/tAMPK	Two-way ANOVA<br>Treatment<br>Sex<br>Treatment x Sex<br>Treatment<br>Sex<br>Treatment x Sex<br>Treatment<br>Sex<br>Treatment x Sex	 F(2,32) = 0.1577<br>F(1,32) = 0.04786<br>F(2,32) = 1.269<br>F(2,31) = 0.2182<br>F(1,31) = 8.214<br>F(2,31) = 3.228<br>F(2,30) = 1.569<br>F(1,30) = 5.389<br>F(2,30) = 1.266	 p=0.8547<br>p=0.8282<br>p=0.2947<br>p=0.8052<br>p=0.0074<br>**p=0.0533**<br>p=0.2248<br>p=0.0272<br>p=0.2965	 0.00976<br>0.00149<br>0.0735<br>0.0139<br>0.2094<br>**0.1723**<br>0.0947<br>0.1523<br>0.0779

Figure 9B	Two-way ANOVA			
pAMPK	Treatment	F(2,31) = 0.06162	p=0.9404	0.00396
	Sex	F(1,31) = 2.561	p=0.1196	0.0763
	Treatment x Sex	F(2,31) = 1.681	p=0.2027	0.0979
tAMPK	Treatment	F(2,31) = 1.317	p=0.2825	0.0783
	Sex	F(1,31) = 0.1926	p=0.6648	0.0062
	Treatment x Sex	F(2,31) = 1.018	p=0.3732	0.0616
pAMPK/tAMPK	Treatment	F(2,31) = 1.003	p=0.3784	0.0608
	Sex	F(1,31) = 1.889	p=0.1792	0.0574
	Treatment x Sex	F(2,31) = 0.5864	p=0.5624	0.0364
Figure 9C	Two-way ANOVA			
pAMPK	Treatment	F(2,32) = 0.2176	p=0.8056	0.0134
	Sex	F(1,32) = 0.3811	p=0.5414	0.0118
	Treatment x Sex	F(2,32) = 0.3608	p=0.6999	0.0221
tAMPK	Treatment	F(2,32) = 1.141	p=0.3322	0.0666
	Sex	F(1,32) = 0.4297	p=0.5168	0.0132
	Treatment x Sex	F(2,32) = 1.602	p=0.2172	0.0910
pAMPK/tAMPK	Treatment	F(2,32) = 0.793	p=0.4612	0.0472
	Sex	F(1,32) = 1.245	p=0.2728	0.0374
	Treatment x Sex	F(2,32) = 2.157	p=0.1322	0.1188
Figure 9D	Two-way ANOVA			
pAMPK	Treatment	F(2,32) = 0.3444	p=0.7112	0.0211
	Sex	F(1,32) = 0.07341	p=0.7882	0.0023
	Treatment x Sex	F(2,32) = 2.776	**p=0.0773**	**0.1478**
tAMPK	Treatment	F(2,32) = 0.321	p=0.7277	0.0197
	Sex	F(1,32) = 3.241	**p=0.0813**	**0.0920**
	Treatment x Sex	F(2,32) = 4.096	p=0.0261	0.2038
pAMPK/tAMPK	Treatment	F(2,32) = 0.1382	p=0.8714	0.0086
	Sex	F(1,32) = 0.5035	p=0.4831	0.0155
	Treatment x Sex	F(2,32) = 0.4055	p=0.6700	0.0247
Figure 10A	Two-way ANOVA			
pAMPK	Treatment	F(2,25) = 0.4995	p=0.6127	0.0384
	Sex	F(1,25) = 0.9859	p=0.3303	0.0379
	Treatment x Sex	F(2,25) = 0.6118	p=0.5503	0.0466
tAMPK	Treatment	F(2,27) = 1.165	p=0.3270	0.0794
	Sex	F(1,27) = 4.874	p=0.0359	0.1529
	Treatment x Sex	F(2,27) = 0.695	p=0.5078	0.0489
pAMPK/tAMPK	Treatment	F(2,25) = 0.0852	p=0.4386	0.0638
	Sex	F(1,25) = 2.856	p=0.1034	0.1025
	Treatment x Sex	F(2,25) = 0.00245	p=0.9976	0.0002
Figure 10B	Two-way ANOVA			
pAMPK	Treatment	F(2,30) = 0.3171	p=0.7303	0.0207

	Sex	F(1,30) = 1.184	p=0.2853	0.0380
	Treatment x Sex	F(2,30) 0.4754	p=0.6263	0.0307
tAMPK	Treatment	F(2,30) = 3.236	**p=0.0534**	**0.1775**
	Sex	F(1,30) = 0.9416	p=0.3396	0.0304
	Treatment x Sex	F(2,30) = 0.4460	p=0.6443	0.0289
pAMPK/tAMPK	Treatment	F(2,30) = 0.4034	p=0.6716	0.0262
	Sex	F(1,30) = 0.4794	p=0.4940	0.0157
	Treatment x Sex	F(2,30) = 0.2953	p=0.7465	0.0193
Figure 10C	Two-way ANOVA			
pAMPK	Treatment	F(2,28) = 3.298	**p=0.0517**	**0.1906**
	Sex	F(1,28) = 0.3147	p=0.5793	0.0111
	Treatment x Sex	F(2,28) = 0.2950	p=0.7468	0.0206
tAMPK	Treatment	F(2,29) = 2.287	p=0.1196	0.1362
	Sex	F(1,29) =	p=0.9828	0.000016
	Treatment x Sex	0.0004716	p=0.6359	0.0307
pAMPK/tAMPK	Treatment	F(2,29) = 0.4599	p=0.5765	0.0386
	Sex	F(2,28) = 0.5618	p=0.6349	0.0082
	Treatment x Sex	F(1,28) = 0.2304	p=0.9236	0.0057
		F(2,28) = 0.07974		
Figure 10D	Two-way ANOVA			
pAMPK	Treatment	F(2,32) = 1.231	p=0.3056	0.0714
	Sex	F(1,32) = 1.210	p=0.2796	0.0364
	Treatment x Sex	F(2,32) = 0.02928	p=0.9712	0.0018
tAMPK	Treatment	F(2,32) = 1.754	p=0.1894	0.0988
	Sex	F(1,32) = 1.598	p=0.2153	0.0476
	Treatment x Sex	F(2,32) = 0.9478	p=0.3982	0.0559
pAMPK/tAMPK	Treatment	F(2,31) = 1.077	p=0.3530	0.0650
	Sex	F(1,31) = 2.941	p=0.0963	0.0866
	Treatment x Sex	F(2,32) = 2.006	p=0.1517	0.1145

Footnote: Significant effects as demonstrated by $p<0.05$ are indicated by underlining whereas trend-level results with $p>0.05$ but $p<0.10$ are indicated by bolding.
